# STING cg16983159 methylation: a key factor for glioblastoma immunosuppression

**DOI:** 10.1038/s41392-022-01093-w

**Published:** 2022-07-11

**Authors:** Lei Qiu, Yang Meng, Junhong Han

**Affiliations:** Research Laboratory of Tumor Epigenetics and Genomics, Frontiers Science Center for Disease-related Molecular Network, State Key Laboratory of Biotherapy and Cancer Center, and National Clinical Research Center for Geriatrics, West China Hospital, Sichuan University, 610041 Chengdu, P.R. China

**Keywords:** Tumour immunology, Epigenetics

**Fig. 1:**
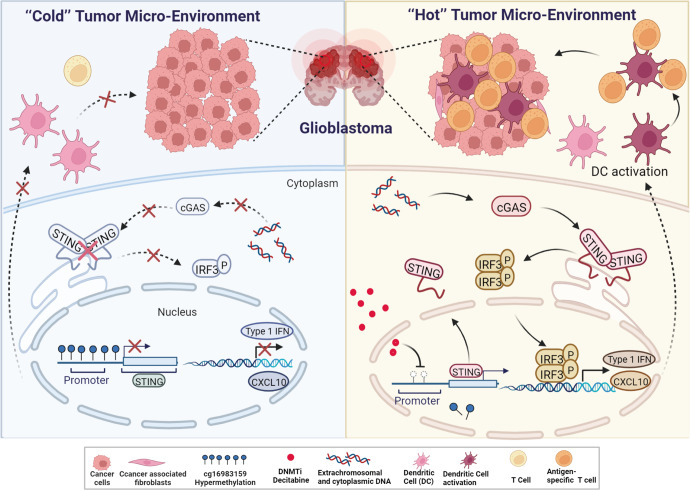
Schematic diagram demonstrating the epigenetic mechanism regulating glioblastoma tumor micro-environment. Left, in glioblastoma, cg16983159 of STING promoter is hypermethylated, silencing the cGAS–STING signaling pathway, suppressing the expression of Type I IFN and CXCL10, thus leading to the “cold” tumor micro-environment. Right, when treated with DNMTi decitabine, cg16983159 of STING promoter is demethylated, turning on STING expression and cGAS–STING signaling, sensitizing GBM cells to immunotherapies. The figure is created with BioRender.com

In a recent study published in *Cancer Cell*, Low et al. shed a light on a novel DNA methylation pattern in the promoter region of the stimulator of interferon genes (STING), which may be a key factor for the “cold” tumor micro-environment (TME) of glioblastoma (GBM), contributing to its immunosuppression.^1^ They have demonstrated that STING expression is epigenetically silenced by cg16983159 methylation in glioma cells as well as normal brain cells and this silencing can be rescued by DNA methyltransferase (DNMT) inhibition (Fig. 1).^1^

Cancer cells, especially those treated with radiation or chemotherapy, often contain high levels of cytosolic DNA.^2^ The cGAS–STING signaling axis is the major sensor of cytosolic DNA and triggers the innate immune response.^3^ Cyclic GMP-AMP (cGAMP) produced by cGAMP synthase (cGAS) binds to STING and activates STING signaling, leading to the production of a series of pro-inflammatory cytokines, such as type I interferons (IFNs) (Fig. 1).^3^ Pro-inflammatory cytokine production and T-cell infiltration are important features of the so-called “hot” TME (T-cell inflamed), whereas the “cold” tumors (non-T-cell inflamed) are characterized by T-cell absence. The “hot” tumors are generally more responsive to immunotherapy, many studies thus focus on converting “cold” TME into “hot” TME in an attempt to achieve a better immunotherapy response.^2^ Earlier studies revealed that cGAS expression in tumor cells and STING activation in dendritic cells (DCs), tumor associate macrophages (TAMs), or endothelial cells are crucial for appropriate type I IFN production as well as CD8 T-cell infiltration into the tumor.^2^ However, by using single-cell RNA-sequencing profiles and Illumina methylation arrays, Low et al. found that although myeloid, endothelial and stromal cells still expressed STING in GBM patients, epigenetic silencing of STING expression in GBM cells alone had led to a “cold” TME, which made GBM suppressive to immunotherapy.^1^

In this study, the authors compared STING gene methylation level on 64 GBM patient samples with samples from normal brain. The CpG site cg16983159 in the STING promoter region was consistently hypermethylated, suggesting that STING mRNA expression was suppressed by cg16983159 methylation. Interestingly, cg16983159 was also hypermethylated in normal brains, indicating that STING promoter hypermethylation is not a result of tumorigenesis, but rather a consistent feature throughout brain development and gliomagenesis.

By sequencing two glioma cell lines treated with bisulfite, the authors observed methylation at most of the 13 CpG sites near the cg16983159 site. Treating cells with decitabine led to demethylation at cg16983159 and upstream sites. Decitabine is a potent DNMT inhibitor (DNMTi) that traps DNMTs on DNA, thus depleting the DNMT pool. Decitabine treatment suppressed cg16983159 methylation, increasing STING expression (Fig. 1). Treating cell lines that were previously unresponsive to STING signaling with decitabine activated innate immune response as well as IFN-induced genes.

GBM is the most frequent and aggressive primary malignant adult tumor in the central nervous system, with rapid growth and frequent relapse.^4^ It is resistant to all currently available standard treatments, with a 5-year overall survival of only 6.8%.^4^ Immunotherapy has dramatically improved the outcome of many patients with advanced solid tumors, but sadly has not yet been able to translate into improving prognosis for GBM patients.^4^ Studies of GBM have demonstrated resistance mechanisms at all phases (intrinsic resistance, adaptive resistance, and acquired resistance) of the antitumor immune response.^5^

Current immunotherapy for GBM includes DC-based vaccines, peptide vaccines, checkpoint inhibitors, chimeric T-cell receptors, and oncolytic virotherapy.^4^ Due to the immunosuppression of GBM, novel mechanisms that sensitize GBM to immunotherapies are being vigorously studied. It is odd for GBM to be immunosuppressive and have a “cold” TME, since GBM frequently carries cytoplasmic and extrachromosomal DNA, which normally should trigger the cGAS–STING signaling pathway.^1^ By demonstrating the epigenetic silencing of STING in GBM cells and the immune reactivation by DNMTi decitabine, this study by Low et al. certainly inspires for new combinatorial therapeutic strategies. Several issues must be solved though before DNMTis can be actually used in clinical treatment. First, although Low et al. demonstrated effective activation of the cGAS–STING pathway by decitabine treatment in cell lines, many potential immunotherapies seemed to fail in clinical trials in GBM later on, with few patients showing durable responses.^4^ The DNA incorporating functional feature of DNMTis make them advantageous in targeting the rapidly dividing cells, which is the case for cultured cell lines. However, in the actual TME, not all cells are actively dividing, which may attenuate the efficacy of DNMTis. Further studies are definitely needed to demonstrate efficacy of decitabine against GBM in animal models and clinical trials. Another concern is the temporary effect of currently available DNMTis, whose withdrawal often leads to recovered DNA methylation levels, thus efforts should be put in developing therapies that would have lasting effect on the demethylation of STING. Moreover, DNMTs usually target multiple regions on the genome, it is thus necessary to determine potential side effects in clinical application of pan DNMTis. Last but not least, many drugs against GBM face a big challenge in clinical efficacy, as the blood-brain barrier is a major limitation for drug delivery. Many studies have been focusing on overcoming this barrier, it is thus important to consider effective drug delivery methods in future application of DNMTis in GBM treatment.

Taken together, this pioneering work by Low et al. demonstrates a novel epigenetic regulatory mechanism in GBM, along with other brain tumors and neuroectoderm-derived tumors, where they find shared cg16983159 hypermethylation in STING promoter in multiple cancers and their corresponding normal tissues of origin. Undoubtedly, the discovery of cg16983159 hypermethylation in GBM immunosuppression provides a new approach for combinatorial anti-cancer treatment. Promisingly, DNMTis may be used as potential candidates in clinical applications in treating immunosuppressive GBMs as well as cancers that share the same STING silencing mechanism.
